# Experimental, comparative, duplex mapping study of arterial flow distribution in ischemia and reperfusion by retrograde circulation

**DOI:** 10.1590/1677-5449.190052

**Published:** 2020-07-31

**Authors:** Cesar Roberto Busato, Carlos Alberto Lima Utrabo, Leandro Cavalcante Lipinski, Marcelo de Oliveira Dreweck, Ana Carolina Viezzer Fernandes, Gabriel Sviercoski

**Affiliations:** 1 Universidade Estadual de Ponta Grossa – UEPG, Departamento de Medicina, Ponta Grossa, PR, Brasil.

**Keywords:** venous arterialization, ischemia, reperfusion

## Abstract

Treatment options for critical lower limb ischemia in the absence of the distal bed are limited. Diverting blood flow in a retrograde direction through the venous circulation is one alternative option that is supported by evidence from several published articles. Duplex scanning was used to compare the distribution of arterial flow in hind limbs of pigs maintained in physiological circulation to contralateral limbs subjected to ischemia and reperfusion by retrograde circulation. Flow in limbs with physiological and retrograde circulation was evaluated by duplex scanning with analysis of Peak Systolic Velocity (PSV), End Diastolic Velocity (EDV), and the Resistivity Index (RI) for selected arteries. This comparative analysis of extremities maintained in physiological circulation in relation to those subjected to ischemia and reperfusion by retrograde circulation showed, via duplex scanning, that changes in spectral wave patterns and hemodynamic variables are satisfactory indicators and suggest good distribution of distal blood flow.

## INTRODUCTION

When critical ischemia is present without a distal arterial bed, it is impossible to divert blood to a patent artery in the extremity distal to the obstruction. Diverting blood flow in a retrograde direction via the venous circulation is a feasible option that is supported by evidence from several published studies.^1^^-^9 The underlying concept is based on the theory that if primary arterial blood pressure is absent or greatly reduced in the arterioles, then blood supplied via the arterialized distal venous system will be able to supply peripheral tissues with sufficient oxygenation.^3^^-^5^,^^10^

Studies have attempted to compare, using physical and laboratory variables, the degree of ischemia and reperfusion produced by arterialization of veins in animal models.^11^ This study compared the distribution of arterial vascular flow in limbs in which physiological circulation was maintained against the flow in contralateral limbs subjected to arterialization of veins, by studying hemodynamic variables and Doppler indices in the vascular beds.

This project was approved by the Animal Research Ethics Committee (CEUA – 024/2017) at the Universidade Estadual de Ponta Grossa (UEPG), Ponta Grossa, PR, Brazil. An observational study was conducted using four Large White and Landrace cross pigs, that had not yet undergone any prior surgical procedures, but were scheduled to be used in practical Operating Technique classes and were used for the experiments before those surgical procedures were undertaken. The animals were premedicated with ketamine (14 mg/kg), xylazine (0.2 mg/kg), and acepromazine (0.4 mg/kg). Anesthesia was induced with propofol (5 mg/kg) and maintained with inhaled isoflurane at a minimum alveolar concentration of 1.2 to 1.7%.

**Figure 1:**
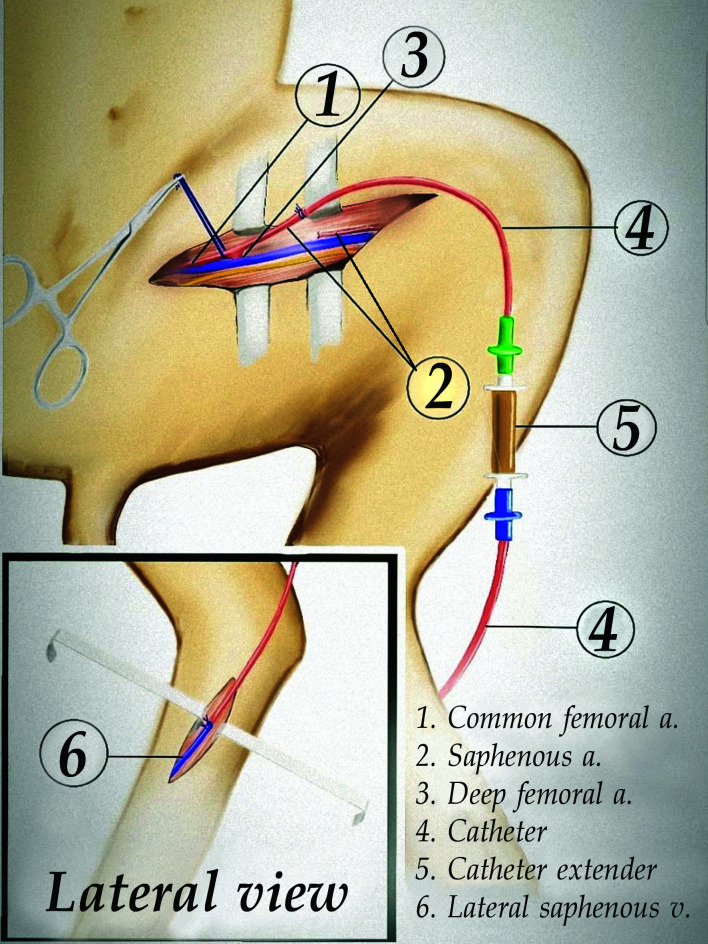
Diagram illustrating the surgical procedure.

The right rear limbs of the animals studied were maintained with physiological circulation, but the left rear limbs subjected to ischemia and reperfusion by arterialization of veins. The left lateral saphenous veins were dissected. After systemic anticoagulation with 5,000 UI of heparin, proximal ligation, longitudinal venotomy, and downstream valvotomy using a Lengua valvotome were performed. The distal extremities were dilated using heparinized saline via a nº 4 catheter, with catheterization, attachment, and closure of the catheters after local administration of 10 mL of heparinized saline (5,000 UI/500 mL of physiological saline). The left common femoral arteries were dissected, with ligature of the distal extremities. The proximal extremities were catheterized with nº 6 catheters, which, after attachment and local administration of 10 mL of heparinized saline, were immediately connected to the lateral saphenous veins using silicone catheters (20 cm Luer Lock Reversible Catheter Extender, double male – 10F; Hartmann^®^, Rio de Janeiro, Brazil), thereby achieving arterialization of the extremity veins. The mean duration of ischemia from ligature of the distal femoral artery to establishment of retrograde circulation was 20 minutes (Figure 1).

Vascular flow in the physiological and arterialized extremities of the limbs was monitored using duplex mapping. These examinations were unblinded and were all conducted by the same radiologist, without cross-checking, using a Samsung/Medison SonoAce R7^®^ scanner (South Korea) with an LN5-12 8MHz linear transducer, lubricated with Sonic-Plus^®^ electroconductive ultrasound gel and positioned in the regions of the chosen vascular beds, the common femoral, saphenous, and medial plantar arteries. In Doppler spectral color mode, the manufacturer’s presets for vascular peripheral studies were used, providing arterial results for Peak Systolic Velocity (PSV), End-Diastolic Velocity (EDV), and spectral waveform (WF). These parameters were used to calculate the Doppler Resistivity Index (RI). The mean time between establishment of retrograde circulation and completion of readings from the right and left rear limbs was 60 minutes. After the experimental procedures and the Operating Technique classes had been completed, the animals were euthanized in accordance with Federal Veterinary Medical Council resolution 1000/2012 (Conselho Federal de Medicina Veterinária - CFMV).

Hemodynamic variables and Doppler indices were measured for each animal and results from limbs with physiological circulation were compared to results for contralateral arterialized limbs (Table 1). For the purposes of comparison, ratios were calculated for arterialized limbs against physiological limbs for the variables described above. This procedure provides an index for each variable expressing the result for the arterialized limb as a proportion of the result for the physiological limb of the same animal (Table 1).

**Table 1:** Proportions obtained by calculating ratios for animals’ arterialized/physiological limbs in the selected vascular beds and their respective means.

	**PSV (A/P)**	**EDV (A/P)**	**RI (A/P)**
**Medial plantar artery**			
Pig 1	0.378536585	1.108571429	0
Pig 2	0.798532355	1.278350515	0.355077412
Pig 3	1.236657737	2.907750832	0.626229168
Pig 4	1.109047619	1.638185654	0.607377988
Mean	0.880693574	1.733214608	0.397171142
**Saphenous artery**			
Pig 1	0.835980861	0.329371817	1.497646945
Pig 2	1.167390578	0.705561614	1.17474589
Pig 3	0.708371665	1.533568905	0.693541504
Pig 4	0.425147183	0.630193906	0.671957036
Mean	0.784222572	0.79967406	1.009472844
**Femoral artery**			
Pig 1	0.697104677	0.451960784	1.103369824
Pig 2	0.871744694	0.542525773	1.049950646
Pig 3	1.265533411	1.7	0.926154743
Pig 4	0.955450557	0.660446518	1.157368548
Mean	0.947458335	0.838733269	1.05921094

PSV (A/P) = arterialized/physiological limb ratio for peak systolic velocity; EDV (A/P) = arterialized/physiological limb ratio for end-diastolic velocity; RI (A/P) = arterialized/physiological limb ratio for Doppler resistivity index .

The Doppler study of arterial blood flow in the arterialized limbs demonstrated equivalence between the triphasic and biphasic wave patterns in the femoral artery, predominance of the biphasic pattern in the saphenous artery, and predominance of the monophasic pattern in the medial plantar artery (Table 2). The small sample size resulted in highly heterogeneous data, which made statistical analysis impossible. This diversity was primarily detected in the coefficient of variation for EDV in the vascular beds studied.

**Table 2:** Waveforms observed in the selected arterial beds and their proportions (%) in the animals’ physiological and arterialized limbs.

		Waveform					
**Artery**		**Triphasic**		**Biphasic**		**Monophasic**	
	Physiological	2	50.00%	2	50.00%	0	0.00%
Femoral	Arterialized	2	50.00%	2	50.00%	0	0.00%
	
	Physiological	0	0.00%	4	100.00%	0	0.00%
Saphenous	Arterialized	0	0.00%	3	75.00%	1	25.00%
		
	Physiological	0	0.00%	4	100.00%	0	0.00%
Medial plantar	Arterialized	0	0.00%	1	25.00%	3	75.00%

While the best parameter to use to evaluate the circulation of a limb is its contralateral counterpart, measurements are taken sequentially, so the hemodynamic conditions are not identical. Use of inelastic catheters with calibers smaller than the vessels may increase resistivity, while bypass to the lower pressure venous and arterial systems, by ligature of the femoral, may reduce it. Therefore, the numerical result of this equation may exhibit discrepancies.

**Figure 2:**
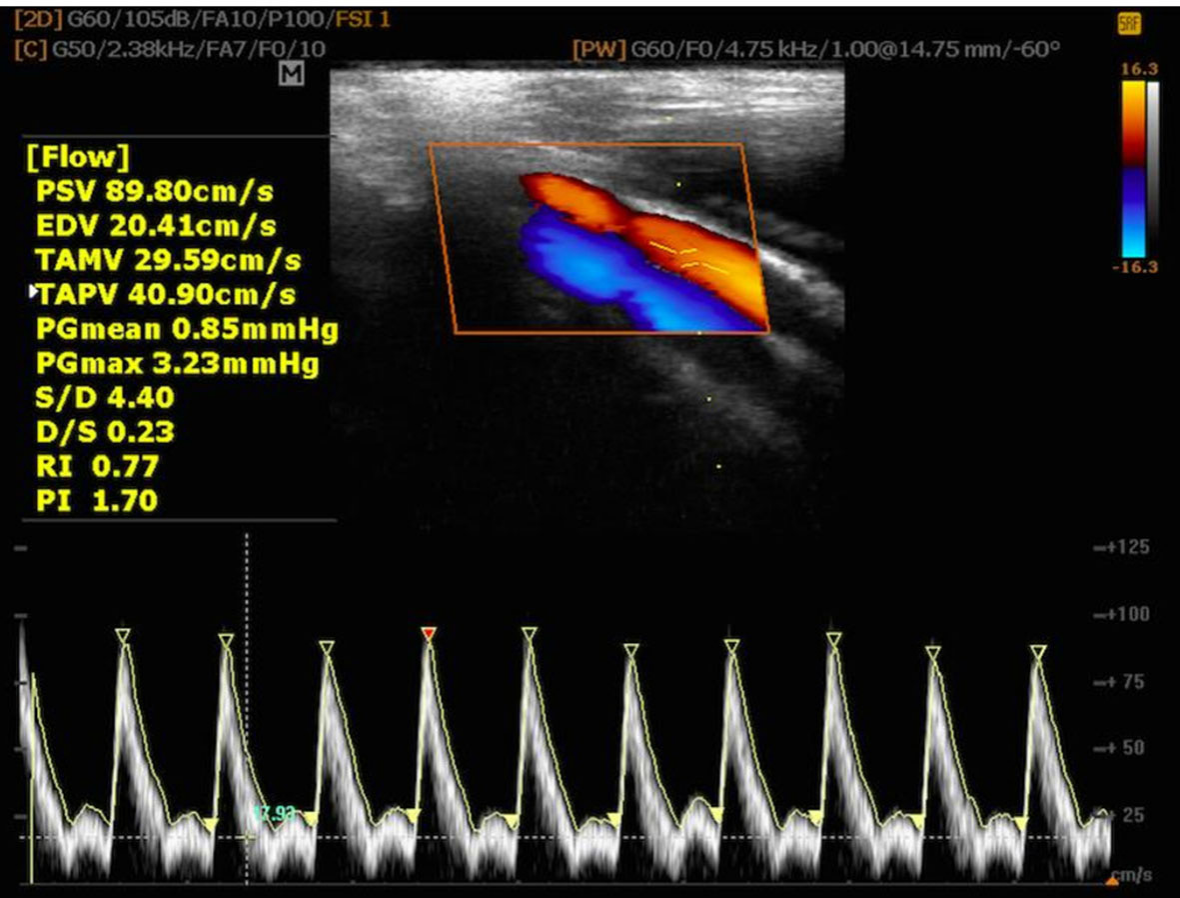
Triphasic spectral waveform in femoral artery of the limb with physiological circulation maintained. PSV = peak systolic velocity; EDV = end-diastolic velocity; TAMV = time-averaged mean velocity; TAPV = time-averaged peak velocity; PGmean = mean pressure gradient; PGmax = maximum pressure gradient; S/D = PSV/EDV; D/S = EDV/PSV; RI = resistivity index; PI = pulsatility index.

The hemodynamic variables used in this study were chosen to quantitatively evaluate the Doppler spectrum. When viewing an arterial spectral wave (Figure 2), the maximum point reflects PSV, i.e., the highest velocity achieved in the vascular bed during a cardiac cycle; while the minimum point in the shape of the wave gives the value of EDV.^12^^,^^13^

In turn, the Doppler indices provide data on peripheral vascular resistance based on the variables defined above (PSV, EDV). The RI was described by Pourcelot in 1974 and is determined by subtracting EDV from PSV and dividing the result by PSV (RI = PSV – EDV/PSV).^12^^,^^13^

Changes in hemodynamic indices are of help for identifying disorders that affect tissue perfusion or complacency of the vascular beds. Several factors in addition to resistance to blood flow through the peripheral vessels influence the values of Doppler indices, including heart rate, blood pressure, and length and elasticity of vessels,^12^ highlighting the importance of comparing the physiological and arterialized sides of the same animal, as described in the literature.^14^^,^^15^ Although the dissections and catheterizations performed to create retrograde circulation were conducted on the left, it is probable that the right limb also underwent changes to blood flow, provoked by changes to peripheral resistance on the left.

Although ultrasound readings on the left were taken upstream of the point of ligature of the common femoral artery, the limbs with retrograde circulation may have exhibited changes in relation to the physiological side, as a function of the variations in peripheral resistance observed (Table 1 and Table 2). In one of the saphenous arteries, a monophasic Doppler WF and reduced PSV and EDV were observed together with maintenance of RI, suggesting a retrograde circulation scenario (Table 2).

In the distal arterial beds (medial plantar artery), the expectation was that RI would be reduced, demonstrating presence of post-arterialization flow. The results showed that this monophasic flow was present, according to the RI data (Table 1 and Table 2).

Triphasic WF (Figure 2) is typical of vascular beds with elevated RI and is represented graphically by tapered systolic peaks and reverse blood flow at the start of diastole. Reverse diastolic blood flow occurs in these vessels because the elevated PSV is reflected with high impedance by the peripheral vascular bed, but, as the vascular diameter returns to normal, diastolic blood flow becomes continuous.^12^

**Figure 3:**
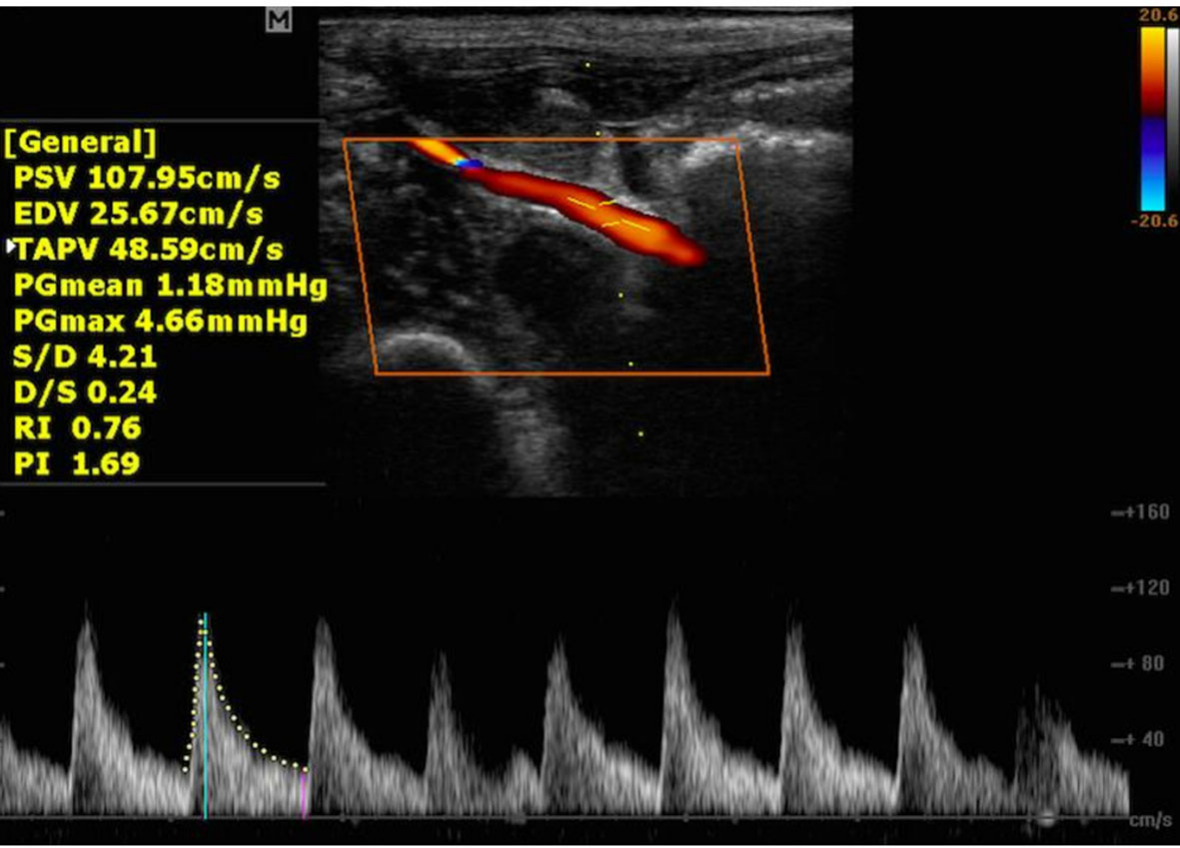
Biphasic spectral waveform in femoral artery of the limb with arterialized circulation. PSV = peak systolic velocity; EDV = end-diastolic velocity; TAPV = time-averaged peak velocity; PGmean = mean pressure gradient; PGmax = maximum pressure gradient; S/D = PSV/EDV; D/S = EDV/PSV; RI = resistivity index; PI = pulsatility index.

**Figure 4:**
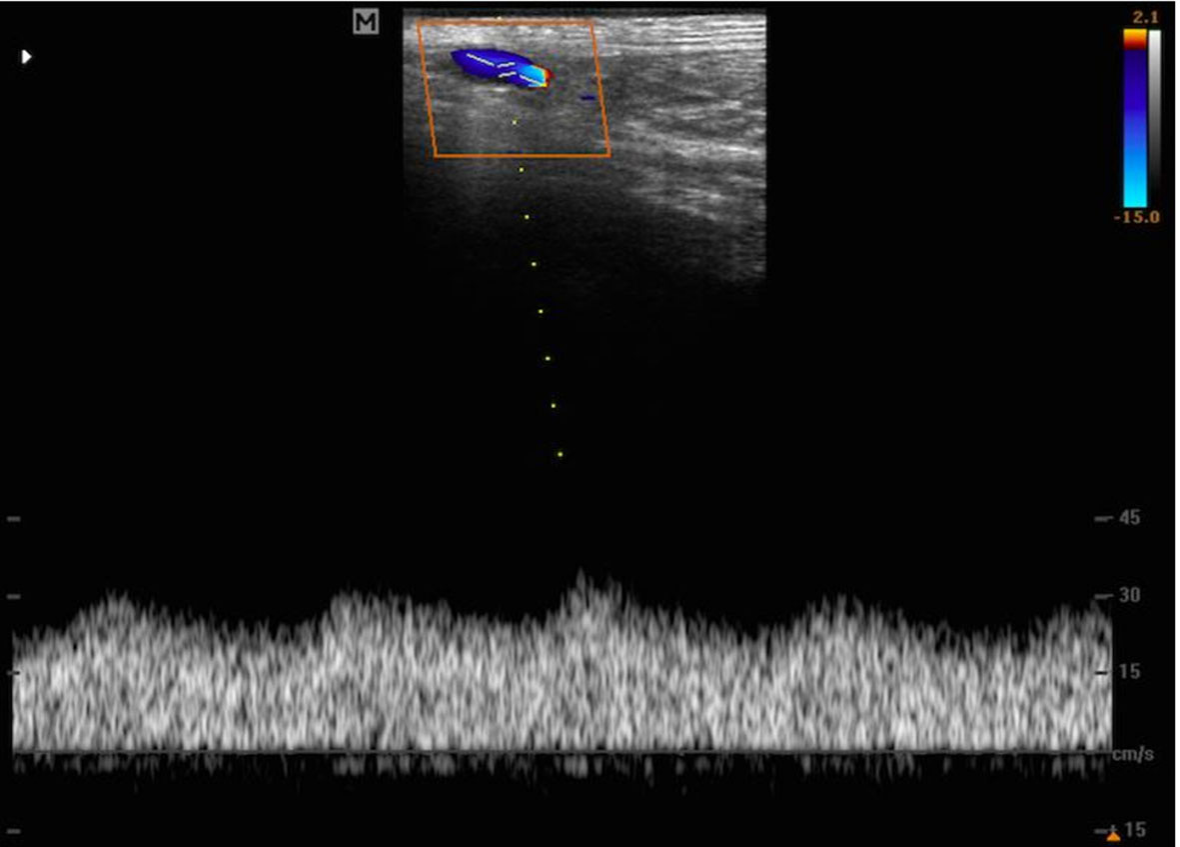
Monophasic spectral waveform in medial plantar artery of the limb with arterialized circulation.

Biphasic WF (Figure 3) is typical of regions where there is loss of high resistivity, as in tissues with post-stenotic circulation. This is characterized by systolic peaks that are also tapered, but are wider than in triphasic WF, and by continuous diastolic blood flow, without reverse flow.^12^ In turn, monophasic WF (Figure 4) is typically venous or of low resistivity beds, such as in arteriovenous fistulae (low RI), and is generally laminar.^12^

In the physiological arterial beds, the WF in the common femoral artery was equivalently distributed between the triphasic and biphasic forms, while biphasic waveforms predominated in the other arteries. In the arterialized vessels, changes in WF were observed in one saphenous artery and three medial plantar arteries with a predominance of monophasic waves, characterizing a change to a low resistivity pattern compatible with arterialized blood flow. The importance of analyzing WFs in the context of arterialization of veins is to identify changes so that it can be suggested that blood flow in the vascular beds originates from a different route than its physiological origin.

Possible anatomic biases could have been reduced by randomizing which side would be arterialized. Preoperative measurements, which were not taken, could have been used to detect any hemodynamic changes that took place on the physiological side after arterialization of contralateral veins. Measuring the ankle-brachial index, before and after arterialization of veins could have provided support for any changes that would have been detected by duplex mapping.

Comparative analysis of extremities in which physiological circulation was maintained in comparison to those subjected to ischemia and reperfusion by retrograde circulation showed that, in the latter, spectral waveforms and hemodynamic variables obtained with duplex mapping were satisfactory indicators and suggested good distribution of blood flow in the vascular beds. However, additional studies are needed to evaluate Doppler ultrasonography parameters in the vessels involved with greater precision.
