# Supplementary material for: Experimental, comparative, duplex mapping study of arterial flow distribution in ischemia and reperfusion by retrograde circulation
Source: J Vasc Bras. 2020 Jul 31;19:e20190052. doi: 10.1590/1677-5449.190052 (PMC8202190; doi:10.1590/1677-5449.190052)
Supplement: Translation

# Translation: Estudo comparativo experimental da distribuição do fluxo arterial, em isquemia e reperfusão por circulação retrógrada, através de mapeamento dúplex

**Authors:** Cesar Roberto Busato, Carlos Alberto Lima Utrabo, Leandro Cavalcante Lipinski, Marcelo de Oliveira Dreweck, Ana Carolina Viezzer Fernandes, Gabriel Sviercoski

**Affiliations:** 1 Universidade Estadual de Ponta Grossa – UEPG, Departamento de Medicina, Ponta Grossa, PR, Brasil.

**Keywords:** arterialização venosa, isquemia, reperfusão

## Abstract

A isquemia crítica de membro inferior sem leito distal tem opções restritas para tratamento. Desviar o fluxo sanguíneo de maneira retrógrada através da circulação venosa é uma alternativa amparada em evidências de inúmeros trabalhos publicados. Comparou-se através de mapeamento dúplex a distribuição do fluxo sanguíneo arterial em membros posteriores de suínos mantidos em circulação fisiológica com o contralateral submetido à isquemia e reperfusão por circulação retrógrada. O fluxo nos membros em circulação fisiológica e retrógrada foi avaliado por mapeamento dúplex através da determinação da velocidade de pico sistólico (VPS), da velocidade diastólica final (VDF) e do índice de resistividade (IR) em artérias selecionadas. A análise comparativa das extremidades mantidas em circulação fisiológica em relação às mantidas em isquemia e reperfusão, por circulação retrógrada, mostrou nestas, ao mapeamento dúplex, que as alterações nos padrões de onda espectral e nas variáveis hemodinâmicas são indicadores satisfatórios e sugerem boa distribuição do fluxo sanguíneo vascular distal.

## INTRODUÇÃO

Em isquemia crítica sem leito arterial distal, não é possível derivar o sangue para uma extremidade arterial pérvia distal à obstrução. Desviar o fluxo sanguíneo de maneira retrógrada através da circulação venosa é uma alternativa viável amparada em evidências de inúmeros trabalhos publicados^1^^-^9. Esse conceito baseia-se na teoria de que, na ausência ou diminuição de pressão arterial primária nas arteríolas, o sangue fornecido utilizando o sistema venoso distal por meio da arterialização é capaz de suprir os tecidos periféricos e fornecer oxigenação adequada^3^^-^5^,^^10^.

Trabalhos procuraram comparar, através de variáveis físicas e laboratoriais, o grau de isquemia e a reperfusão produzida por arterialização venosa em modelos animais^11^. Este trabalho comparou a distribuição do fluxo vascular arterial em membros mantidos em circulação fisiológica com contralaterais submetidos à arterialização venosa, através da medida de variáveis hemodinâmicas e índices Doppler nos leitos vasculares.

Este projeto foi aprovado pelo Comitê de Ética em Pesquisa Animal (CEUA – 024/2017) da Universidade Estadual de Ponta Grossa (UEPG), Ponta Grossa, PR, Brasil. Foi realizado um estudo observacional, em que foram utilizados quatro suínos cruzados Large White e Landrace, livres de qualquer procedimento cirúrgico prévio, destinados às aulas práticas de Técnica Operatória antes dos procedimentos cirúrgicos. Os animais receberam medicação pré-anestésica com ketamina (14 mg/kg), xilazina (0,2 mg/kg) e acepromazina (0,4 mg/kg). Foram induzidos à anestesia com propofol (5 mg/kg) e mantidos em anestesia inalatória com isofluorano em concentração alveolar mínima de 1,2 a 1,7%.

Membros posteriores direitos dos animais estudados foram mantidos em circulação fisiológica enquanto membros posteriores esquerdos foram submetidos à isquemia e reperfusão por arterialização venosa. Realizaram-se, à esquerda, dissecções de veias safenas laterais. Após anticoagulação sistêmica com 5.000 UI de heparina, procederam-se ligaduras proximais, venotomias longitudinais e rotura das válvulas a jusante com auxílio de valvulótomo de Lengua. Foi feita a dilatação das extremidades distais com soro heparinizado através de sonda nº 4 e cateterização, fixação e fechamento das sondas após aplicação local com 10 mL de soro heparinizado (5.000 UI/500 mL de soro fisiológico). As dissecções das artérias femorais comuns esquerdas com ligadura das extremidades distais foram realizadas. As extremidades proximais foram cateterizadas com sondas nº 6, as quais, após fixação e aplicação local com 10 mL de soro heparinizado, foram imediatamente conectadas às veias safenas laterais utilizando-se cateteres de silicone (extensor para Cateter Reversível Luer Lock 20 cm, 2 conectores macho – 10F; Hartmann®, Rio de Janeiro, Brasil), estabelecendo-se a arterialização venosa da extremidade. O tempo médio de isquemia entre a ligadura da artéria femoral distal e o estabelecimento da circulação retrógrada foi de 20 minutos (Figura 1).

**Figura 1:** Desenho esquemático do procedimento cirúrgico.

A avaliação do fluxo vascular nas extremidades fisiológica e arterializada dos membros foi realizada através do exame de mapeamento dúplex. O exame foi realizado de forma aberta, sempre pelo mesmo radiologista, sem contraconferência, em um aparelho de ultrassom Samsung/Medison SonoAce R7® (Coreia do Sul) com transdutor linear modelo LN5-12 de frequência 8MHz, lubrificado com gel eletrocondutor para ultrassom Sonic-Plus® e posicionado na região dos leitos vasculares eleitos, as artérias femoral comum, safena e plantar medial. No modo de medição Doppler espectral e colorido, utilizou-se o *preset* do fabricante para estudo vascular periférico, do qual se obteve resultados arteriais de velocidade de pico sistólico (VPS), velocidade diastólica final (VDF) e padrão de onda espectral (PO). Sobre esses, foi calculado o índice Doppler de resistividade (IR). O tempo médio entre o estabelecimento da circulação retrógrada e o final da leitura nas artérias dos membros posteriores direito e esquerdo foi de 60 minutos. Ao final dos procedimentos do experimento e das aulas de Técnica Operatória, os animais foram submetidos à eutanásia conforme resolução 1000/2012 do Conselho Federal de Medicina Veterinária (CFMV).

Cada animal teve as variáveis hemodinâmicas e índices Doppler determinados, e aqueles dos membros com circulação fisiológica foram comparados aos dos membros contralaterais arterializados (Tabela 1). Para efeitos de comparação, foram consideradas as razões entre os membros arterializado e fisiológico das variáveis supracitadas. Assim, tem-se um índice apontando a proporção de uma determinada variável no membro arterializado correspondente à do lado fisiológico do mesmo animal (Tabela 1).

**Tabela 1:** Proporções obtidas através da relação membro arterializado/fisiológico dos animais e leitos vasculares selecionados e suas respectivas médias.

	**VPS (A/F)**	**VDF (A/F)**	**IR (A/F)**
**A. Plantar medial**			
Suíno 1	0,378536585	1,108571429	0
Suíno 2	0,798532355	1,278350515	0,355077412
Suíno 3	1,236657737	2,907750832	0,626229168
Suíno 4	1,109047619	1,638185654	0,607377988
Média	0,880693574	1,733214608	0,397171142
**A. Safena**			
Suíno 1	0,835980861	0,329371817	1,497646945
Suíno 2	1,167390578	0,705561614	1,17474589
Suíno 3	0,708371665	1,533568905	0,693541504
Suíno 4	0,425147183	0,630193906	0,671957036
Média	0,784222572	0,79967406	1,009472844
**A. Femoral**			
Suíno 1	0,697104677	0,451960784	1,103369824
Suíno 2	0,871744694	0,542525773	1,049950646
Suíno 3	1,265533411	1,7	0,926154743
Suíno 4	0,955450557	0,660446518	1,157368548
Média	0,947458335	0,838733269	1,05921094

VPS (A/F) = relação membro arterializado/fisiológico da velocidade de pico sistólico; VDF (A/F) = relação membro arterializado/fisiológico da velocidade diastólica final; IR (A/F) = relação membro arterializado/fisiológico do índice Doppler de resistividade.

A pesquisa de fluxo sanguíneo arterial ao Doppler em membros arterializados demonstrou equivalência entre os padrões de onda trifásicos e bifásicos na artéria femoral, predominância de padrão bifásico na artéria safena e monofásico na artéria plantar medial (Tabela 2). O número amostral reduzido resultou em grande heterogeneidade nos dados coletados, o que impossibilitou a análise estatística. Tal diversidade foi verificada principalmente através do coeficiente de variação da VDF nos leitos vasculares estudados.

**Tabela 2:** Padrões de onda encontrados nos leitos arteriais selecionados e suas proporções (%) nos membros fisiológicos e arterializados dos animais.

		Padrão de onda					
**Artéria**		**Trifásico**		**Bifásico**		**Monofásico**	
	Fisiológico	2	50,00%	2	50,00%	0	0,00%
Femoral	Arterializado	2	50,00%	2	50,00%	0	0,00%
	
	Fisiológico	0	0,00%	4	100,00%	0	0,00%
Safena	Arterializado	0	0,00%	3	75,00%	1	25,00%
		
	Fisiológico	0	0,00%	4	100,00%	0	0,00%
Plantar medial	Arterializado	0	0,00%	1	25,00%	3	75,00%

Embora o melhor parâmetro para avaliar a circulação em um membro seja o seu contralateral, as determinações são realizadas de maneira sequencial de tal forma que as condições hemodinâmicas não são idênticas. A utilização de cateteres inelásticos de calibres menores aos dos vasos pode aumentar a resistividade enquanto o desvio para o sistema venoso e arterial de menor pressão, pela ligadura da femoral, pode diminui-la. Assim, o resultado dessa equação em números pode apresentar algumas diversidades.

As variáveis hemodinâmicas utilizadas neste estudo têm a finalidade de avaliar quantitativamente o espectro Doppler. Na visualização de uma onda espectral arterial (Figura 2), o ponto máximo corresponde à VPS, ou seja, à máxima velocidade alcançada no leito vascular durante um ciclo cardíaco; o ponto mínimo na morfologia da onda é o valor da VDF^12^^,^^13^.

**Figura 2:** Padrão de onda espectral trifásico em artéria femoral de membro mantido em circulação fisiológica. PSV = peak systolic velocity; EDV = end-diastolic velocity; TAMV = time-averaged mean velocity; TAPV = time-averaged peak velocity; PGmean = mean pressure gradient; PGmax = maximum pressure gradient; S/D = PSV/EDV; D/S = EDV/PSV; RI = resistivity index; PI = pulsatility index.

Já os índices Doppler fornecem dados acerca da resistência vascular periférica por meio dos valores obtidos nas variáveis supracitadas (VPS, VDF). O IR, descrito por Pourcelot em 1974, é determinado através da subtração entre a VPS e a VDF, dividida pela VPS (IR = VPS – VDF/VPS)^12^^,^^13^.

As alterações nos índices hemodinâmicos auxiliam na identificação de desordens que afetem a perfusão tecidual ou a complacência dos leitos vasculares. Diversos fatores além da resistência ao fluxo sanguíneo nos vasos periféricos, como a frequência cardíaca, a pressão sanguínea e o comprimento e a elasticidade dos vasos, influenciam nos valores dos índices Doppler^12^, ressaltando-se a importância da comparação entre os lados fisiológico e arterializado do mesmo animal, como encontrado na literatura^14^^,^^15^. Embora as dissecções e os cateterismos para circulação retrógrada tenham sido realizados à esquerda, é provável que o membro direito tenha sofrido alterações no fluxo sanguíneo, provocadas por modificações da resistência periférica à esquerda.

Embora a leitura pelo ultrassom à esquerda tenha sido feita a montante do ponto de ligadura da artéria femoral comum, os membros em circulação retrógrada poderiam apresentar alterações, em relação ao fisiológico, na dependência da variação da resistência periférica encontrada (Tabela 1 e Tabela 2). Já em uma artéria safena, verificou-se um PO monofásico e redução da VPS e da VDF com manutenção do IR, sugerindo cenário da circulação retrógrada (Tabela 2).

Nos leitos arteriais distais (artéria plantar medial), o esperado era a diminuição dos IRs para poder caracterizar a existência de fluxo pós-arterialização. Os resultados mostraram que esse fluxo monofásico está presente através dos dados do IR (Tabela 1 e Tabela 2).

O PO trifásico (Figura 2) é típico de leitos vasculares com IR elevado e é graficamente representado por picos sistólicos afilados e fluxo sanguíneo reverso no início da diástole. O fluxo sanguíneo diastólico reverso ocorre nesses vasos porque a VPS elevada é refletida com alta impedância pelo leito vascular periférico, mas, à medida que o diâmetro vascular retorna ao normal, o fluxo sanguíneo diastólico torna-se contínuo^12^.

O PO bifásico (Figura 3) é típico de regiões onde há perda da alta resistividade, como em um tecido com circulação pós-estenótica. Caracteriza-se pela presença de picos sistólicos também afilados, porém mais amplos que os PO trifásicos, e fluxo sanguíneo diastólico contínuo, sem fluxo reverso^12^. Já o PO monofásico (Figura 4) é tipicamente venoso ou de leitos com baixa resistividade, como em fístula arteriovenosa (baixo IR), sendo geralmente laminar^12^.

**Figura 3:** Padrão de onda espectral bifásico em artéria femoral de membro arterializado. PSV = peak systolic velocity; EDV = end-diastolic velocity; TAPV = time-averaged peak velocity; PGmean = mean pressure gradient; PGmax = maximum pressure gradient; S/D = PSV/EDV; D/S = EDV/PSV; RI = resistivity index; PI = pulsatility index.

**Figura 4:** Padrão de onda espectral monofásico em leito vascular distal de artéria plantar medial em membro arterializado.

Quanto aos leitos arteriais fisiológicos, o PO na artéria femoral comum foi equivalente na distribuição entre os padrões tri e bifásicos, enquanto o bifásico predominou nas demais artérias. Já nos vasos arterializados, se verificou alterações no PO de uma artéria safena e de três artérias plantares mediais com predominância de ondas monofásicas, caracterizando mudança para um padrão de baixa resistividade compatível com fluxo sanguíneo arterializado. A importância da avaliação dos POs no contexto da arterialização venosa é a identificação da mudança para que se possa sugerir que o fluxo sanguíneo nos leitos vasculares seja proveniente de uma via diferente da fisiológica.

Possíveis vieses anatômicos poderiam ser reduzidos com sorteio do lado a arterializar. Medidas pré-operatórias, que não foram realizadas, poderiam demonstrar possíveis alterações hemodinâmicas, no lado fisiológico, após a arterialização venosa contralateral. A medida de índice tornozelo braquial, pré e pós-arterialização venosa, poderia ter dado suporte para as possíveis alterações que pudessem ser encontradas ao mapeamento dúplex.

A análise comparativa das extremidades mantidas em circulação fisiológica em relação às mantidas em isquemia e reperfusão por circulação retrógrada mostrou nestas, ao mapeamento dúplex, que os padrões de onda espectral e as variáveis hemodinâmicas são indicadores satisfatórios e sugerem boa distribuição do fluxo sanguíneo nos leitos vasculares. No entanto, novos estudos são necessários para se avaliar com maior precisão os parâmetros do eco-Doppler nos vasos envolvidos.
